# Influence of alcohol consumption and alcohol metabolism variants on breast cancer risk among Black women: results from the AMBER consortium

**DOI:** 10.1186/s13058-023-01660-1

**Published:** 2023-06-12

**Authors:** Kristin L. Young, Andrew F. Olshan, Kathryn Lunetta, Mariaelisa Graff, Lindsay A. Williams, Song Yao, Gary R. Zirpoli, Melissa Troester, Julie R. Palmer

**Affiliations:** 1 Department of Epidemiology, Gillings School of Global Public Health, University of North Carolina at Chapel Hill, Chapel Hill, NC 27516 USA; 2 Department of Biostatistics, Boston University School of Public Health, Boston, MA 02118 USA; 3 Division of Epidemiology and Clinical Research, Department of Pediatrics, University of Minnesota, Minneapolis, MN 55455 USA; 4 Department of Cancer Prevention and Control, Roswell Park Comprehensive Cancer Center, Buffalo, NY 14263 USA; 5 Slone Epidemiology Center, Boston University, Boston, MA 02215 USA

**Keywords:** Alcohol, Breast cancer, Gene-environment interaction

## Abstract

**Background:**

Moderate to heavy alcohol consumption is associated with an increased risk of breast cancer. The etiologic role of genetic variation in genes involved in ethanol metabolism has not been established, with little information available among women of African ancestry.

**Methods:**

Our analysis from the African American Breast Cancer Epidemiology and Risk (AMBER) Consortium included 2889 U.S. Black women who were current drinkers at the time of breast cancer diagnosis (N cases = 715) and had available genetic data for four ethanol metabolism genomic regions (*ADH, ALDH, CYP2E1*, and *ALDH2*). We used generalized estimating equations to calculate genetic effects, gene* alcohol consumption (≥ 7drinks/week vs. < 7/week) interactions, and joint main plus interaction effects of up to 23,247 variants in ethanol metabolism genomic regions on odds of breast cancer.

**Results:**

Among current drinkers, 21% of cases and 14% of controls reported consuming ≥ 7 drinks per week. We identified statistically significant genetic effects for rs79865122-C in *CYP2E1* with odds of ER- breast cancer and odds of triple negative breast cancer, as well as a significant joint effect with odds of ER- breast cancer (≥ 7drinks per week OR = 3.92, < 7 drinks per week OR = 0.24, *p*_*joint*_ = 3.74 × 10^−6^). In addition, there was a statistically significant interaction of rs3858704-A in *ALDH2* with consumption of ≥ 7 drinks/week on odds of triple negative breast cancer (≥ 7drinks per week OR = 4.41, < 7 drinks per week OR = 0.57, *p*_*int*_ = 8.97 × 10^–5^).

**Conclusions:**

There is a paucity of information on the impact of genetic variation in alcohol metabolism genes on odds of breast cancer among Black women. Our analysis of variants in four genomic regions harboring ethanol metabolism genes in a large consortium of U.S. Black women identified significant associations between rs79865122-C in *CYP2E1* and odds of ER- and triple negative breast cancer. Replication of these findings is warranted.

**Supplementary Information:**

The online version contains supplementary material available at 10.1186/s13058-023-01660-1.

## Background

Alcohol consumption has consistently been recognized as a risk factor for breast cancer [1, 2]. Previous epidemiologic studies of the association between alcohol consumption and breast cancer risk in the United States have largely been conducted in persons of European ancestry. An analysis of 5108 cases of invasive breast cancer from the African American Breast Cancer Epidemiology and Risk (AMBER) Consortium indicated that African American women who reported current drinking of ≥ 14 drinks per week had higher odds of invasive breast cancer compared with light drinkers (> 0 to < 4 drinks per week) [adjusted OR (AOR) (95% CI) = 1.33 (1.07–1.64)], while those who reported drinking ≥ 7 drinks per week had higher odds of human epidermal growth factor receptor 2 negative (HER2-) breast cancer [AOR (95% CI) = 1.36 (1.09–1.70) [3].

There are many possible biological mechanisms for the association of alcohol consumption and breast cancer, including ethanol metabolism, increased levels of circulating estrogen, cellular proliferation, impact on DNA repair, and interference with the absorption and metabolism of nutrients such as folate and carotenoids [2, 4]. Alcohol metabolism pathways involve two key enzymes: alcohol dehydrogenase (ADH), which oxidizes alcohol to acetaldehyde—a reaction that produces a carcinogen as well as reactive oxygen species [5] that can damage DNA; and aldehyde dehydrogenase (ALDH), which converts acetaldehyde to acetic acid [6]. Other enzymes involved in ethanol metabolism include those in the microsomal ethanol-oxidizing system, Cytochrome P450 2E1 (CYP2E1) and catalase (CAT). CYP2E1 only plays a role in ethanol metabolism for heavy drinkers, while CAT metabolizes only a small proportion of alcohol consumed [6]. ADH is encoded by at least 7 genes in humans, which are clustered together on the long arm of chromosome 4, and, except for *ADH7*, are highly expressed in the liver [7]. ALDH enzymes are encoded by at least 19 genes spread across 12 chromosomes, five of which are highly expressed in liver tissue (*ALDH1L1, ALDH2, ALDH4A1, ALDH6A1,* and *ALDH8A1)* [8], though the two primary enzymes involved in acetaldehyde metabolism during ethanol oxidation are ALDH1 (encoded by *ALDH1A1* on chromosome 9) and ALDH2 (encoded by *ALDH2* on chromosome 12). *CYP2E1* and *CAT* are also highly expressed in liver tissue.

Candidate studies of genes involved in alcohol metabolism have yielded mixed results regarding the association of single nucleotide polymorphisms (SNPs) and risk of breast cancer and the interaction of SNPs and alcohol consumption. Earlier genetic studies found that rs1229984 in *ADH1B* [9] and rs698 in *ADH1C* [10] modify the alcohol association with breast cancer, particularly in pre-menopausal women [10]. Studies of Korean women found a significant interaction for breast cancer risk between rs2031920 (CYP2E1*5) in *CYP2E1* and rs671 in *ALDH2* and alcohol intake [11, 12]. A study on post-menopausal women from the Prostate, Lung, Colorectal and Ovarian (PLCO) Cancer Screening Trial found significant interactions between rs1229984-GG in *ADH1B* and all levels of alcohol intake and risk of breast cancer [13]. Several other large studies, however, indicate that SNPs in *ADH1B*, *ADH1C*, and *CYP2E1* do not modify associations of alcohol intake with breast cancer risk [14–17].

Genome-wide association studies (GWAS) have identified a number of low-risk alleles for breast cancer, though none have directly implicated genes related to alcohol metabolism [18], and generally did not account for alcohol consumption [19–22]. Like many epidemiological studies of breast cancer, most GWAS and candidate gene studies to date have predominantly included individuals of European or Asian ancestry [9, 13, 23–32]. To fill this important gap in knowledge regarding potential disparities, we examined the association of ethanol metabolism pathway genetic variants and SNP-alcohol consumption interactions with odds of breast cancer (overall and among cancers with different hormone receptor status) using data from the African American Breast Cancer Epidemiology and Risk (AMBER) Consortium.

## Methods

The AMBER Consortium is a collaboration among the largest etiologic studies of breast cancer in U.S. Black women. For the present analysis, data were drawn from two case–control studies: the Carolina Breast Cancer Study (CBCS) [33] and the Women’s Circle of Health Study (WCHS) [34, 35]; and the prospective cohort Black Women’s Health Study (BWHS) [36, 37]. All study participants provided informed consent prior to participation, and all studies obtained IRB approval from their respective institutions. A total of 3663 cases and 4687 controls provided either blood or saliva for DNA analysis by the AMBER consortium.

For CBCS, population controls were identified through Division of Motor Vehicles (age < 65 years) or Health Care Financing Administration lists (age ≥ 65). For WCHS, population controls were recruited through random digit dialing and community events. The BWHS was sampled as a nested case–control study from the parent cohort, with controls matched to breast cancer cases on 5-year age group, geographic location, and most recent questionnaire completed [38]. Cases were women diagnosed with incident invasive breast cancer or ductal carcinoma in situ (DCIS). Estrogen receptor (ER), progesterone receptor (PR), and human epidermal growth factor receptor-2 (HER2) oncogene expression status were determined using hospital or cancer registry pathology records.

### Genotyping and quality control

Genotyping of DNA from participants in the BWHS, CBCS, and WCHS for the AMBER Consortium was performed in two phases. In our Phase 1 discovery (2014), genotyping of 6,860 AMBER participants was completed by the Center for Inherited Disease Research (CIDR) at Johns Hopkins University using the Illumina Human Exome Beadchip v1.1. This array includes > 240,000 coding variants: > 4500 variants in genes influencing drug metabolism, > 4400 immune system function variants, > 4500 GWAS variants, > 700 eQTLs, as well as > 200,000 variants from the COSMIC catalog of somatic mutations in cancer. AMBER custom content included 160,440 SNPs in 433 genes in breast cancer relevant pathways (e.g., fibroblast growth factor receptor 2 *(FGFR2)*, steroid hormone metabolism, and vitamin D). Of the 246,519 SNPs genotyped, 231,705 autosomal SNPs passed quality control (QC) (call rates < 0.98, Hardy–Weinberg Equilibrium p < 1 × 10^−4^, or > 2 discordant calls in duplicate samples). 6828 AMBER participants passed QC, including 3130 cases and 3698 controls. Imputation of the genotype data to the 1000 Genomes Phase 1 reference panel was performed by the University of Washington using IMPUTE2 [39, 40]. Measured and imputed genotypes from three AMBER studies (CBCS, BWHS, and WCHS) were combined into a final Phase 1 discovery data set of up to 1847 women, including 597 cases and 1250 controls who reported being current drinkers and had phenotype data. Principal components were calculated using EIGENSOFT [41, 42] based on ~ 42,000 common SNPs. Principal components associated with case status (p < 0.1) after controlling for study, DNA source, and matching variables were included in our analyses [43].

In our Phase 2 validation, genome-wide genotyping of DNA samples from 4085 BWHS, CBCS, and WCHS participants was performed by CIDR using the Illumina Multi-Ethnic Global Array (MEGA) (2,036,060 SNPs) with addition of the panel of custom variants used in Phase 1. The MEGA and custom panel data were then combined for QC and analysis. After exclusion of duplicate samples, prevalent cases, and those missing questionnaire data, genotypes for 3999 AMBER participants passed initial CIDR QC and were sent to the Genetic Analysis Center (GAC) at the University of Washington for additional quality control. Cross-phase concordance was examined using data for 122 participants genotyped for 362,014 SNPs in both phases. The mean concordance across all overlapping samples was 0.9999. The mean concordance across all overlapping SNPs was 0.9999, with just 30 SNPs with > 10% discordance across phases, and 12 SNPs with > 90% discordance. Individuals with missing call rates > 0.03 were excluded from analyses. Genotyped SNPs with call rates < 0.98, Hardy–Weinberg equilibrium p < 1 × 10^−4^, > 1 discordant call in duplicate samples, monomorphic SNPs, and those with MAF < 0.01 across all samples were excluded, leaving 1,182,802 genotyped SNPs. All 3999 Phase 2 samples were then imputed by the GAC using the 1000 Genomes Phase 3v4 cosmopolitan reference panel in IMPUTE2 for SNPs with a minor allele count (MAC) ≥ 2 in either African or European 1000 Genomes super populations, resulting in 34,296,243 imputed variants. For this analysis, measured and imputed genotypes from two AMBER studies (BWHS and WCHS) were combined into a final Phase 2 data set of up to 1042 women, up to 118 invasive cases and 924 controls who reported being current drinkers with phenotype data. CBCS participants genotyped under Phase 2 were not included in the analysis due to a lack of genotyped controls.

### Analytic sample

For analysis, we selected all genotyped and imputed variants in a ± 20 kb window of four known alcohol metabolism genes or clusters (*ADH* on chromosome 4, *ALDH1* on chromosome 9*, CYP2E1* on chromosome 10, and *ALDH2* on chromosome 12), resulting in 18,792 variants in Phase 1 discovery and 46,519 variants in Phase 2 validation after QC exclusions, and up to 23,247 variants in the meta-analysis. Among the 3122 participants who reported being current drinkers at time of diagnosis and had genetic data from either phase, we excluded those with DCIS (n = 172) or for whom the nature of the tumor was unknown (n = 60) and the single CBCS control genotyped in Phase 2 who reported being a current drinker, leaving 2889 participants (715 cases and 2174 controls) for our Phase 1 + Phase 2 meta-analyses.

### Hormone receptor status

In this analysis, we examined only invasive breast cancers, and further subdivided them by hormone receptor status: ER positive (393 cases), ER negative (275 cases), PR positive (321 cases), PR negative (345 cases), HER2 positive (114 cases), HER2 negative (458 cases), triple negative (163 cases), and non-triple negative (465 cases).

### Alcohol intake assessment

Alcohol intake was self-reported for each study via questionnaire. BWHS assessed type of alcohol consumption (drinks per day, week, and month) at baseline, with amount consumed assessed in bi-annual follow-up surveys. CBCS assessed alcohol consumption amount by age range (< 25, 25 to 49, and ≥ 50) for each participant, and WCHS recorded amount of alcohol consumed (drinks per day) for each decade of life. To increase power for GxE analyses, we created a categorical variable representing participants who were consumed < 7 drinks per week or ≥ 7 drinks per week to approximate alcohol exposure following the NIAAA definition of moderate alcohol consumption for women [44]. Additional file 1: Table S1 shows the number of invasive cases and controls for each phase of the AMBER study, as well as the total number of cases and controls.

### Statistical analyses

We first examined the association between alcohol consumption (< 7 vs. ≥ 7 drinks per week) on the odds of breast cancer for each hormone receptor status in the combined sample (Phase 1 + Phase 2), using a logistic regression model adjusted for 10-year age categories and study (Model 1), followed by a model additionally adjusted for age at menarche, body mass index (BMI), parity, smoking status (never, current, or former smoker), menopausal status (pre- or post-), level of education, and duration of oral contraceptive use (Model 2) [3].

Single variant association analyses were conducted separately in each phase assuming an additive genetic model using weighted generalized estimating equations (GEE) logistic regression as implemented in SUGEN to account for relatedness in the sample [45].We estimated SNP main effects, SNP* ≥ 7 drinks per week interaction effects, and joint two degree of freedom (2df) (SNP + SNP*alcohol interaction) effects. The 2df test jointly tests the null hypothesis that both the SNP main effect and SNP* ≥ 7 drinks per week interaction effect are equal to zero [46–50]. Previous GxE GWAS demonstrated the utility of a joint genetic plus interaction test to identify variants where the environmental exposure may not have opposite directions of effect on the outcome, but does show a difference in magnitude of effect between exposure strata [47–49]. We ran case–control models among (1) all women (597 invasive cases and 1250 controls in Phase 1 and 118 invasive cases and 924 controls in Phase2), and (2) each hormone receptor status and all controls. In Phase 1, models were adjusted for age group (by ~ 10-year intervals), study site, geographic region, DNA source, principal components, and a weighted variance estimator to account for relatedness among the ~ 250 2nd degree or closer relatives in Phase 1. Phase 2 models were adjusted for age group (by ~ 10-year intervals), study site, DNA source, and principal components. Phase specific results were excluded if imputation quality (r^2^ < 0.4) or effective sample size among cases (effN_case_ < 10), calculated per SNP as (2*MAF_case_)*(1-MAF_case_)*N_case_*imputation quality. To increase power to detect potential interaction effects (Additional file 2), phase specific results were then meta-analyzed, using a fixed effects inverse variance weighted meta-analysis implemented in METAL [51]. To account for multiple comparisons, we determined the total number of independent variants in each locus using the linkage disequilibrium (LD) pruning function in Plink 1.9 with the following parameters: variant window size = 50, variant window shift = 5, and r^2^ = 0.1. We identified 334 independent variants in the *ADH* locus on chromosome 4, 253 in the *ALDH1* locus on chromosome 9178 in the *CYP2E1* locus on chromosome 10, and 209 in the *ALDH2* locus on chromosome 12 and calculated the p-value threshold required for statistical significance as 0.05/number of independent variants per locus (chr 4: p < 1.5 × 10^–4^, chr 9: p < 1.98 × 10^–4^, chr 10: p < 2.81 × 10^–4^, chr 12: p < 2.39 × 10^–4^). Genetic effects of variants reaching locus-specific statistical significance in either the interaction or joint 2df test were analyzed separately for participants who reported drinking ≥ 7 drinks per week and those who reported drinking < 7 drinks per week.

## Results

In our sample of current drinkers, 21.0% of cases and 13.7% of controls reported consuming ≥ 7 drinks per week. Descriptive characteristics for participants from each study, including details of alcohol consumption, smoking status, use of oral contraceptives, age at menarche, parity, and menopausal status are provided in Additional file 1: Table S1.

Results of our analysis of the association between frequency of alcohol consumption among current drinkers and odds of breast cancer by hormone receptor status are shown in Additional file 1, Table 1. In both age-adjusted (Model 1) and multivariable models (Model 2), heavier alcohol consumption was associated with an increased odds of any invasive breast cancer [multivariable OR (95% CI): 1.30 (1.00–1.68)], ER- breast cancer [OR (95% CI): 1.48 (1.01–2.14)] and PR- breast cancer [OR (95% CI): 1.50 (1.07–2.10)]; the comparable OR for ER + breast cancer was 1.33 (0.98–1.82)).

**Table 1:** Associations between alcohol consumption and breast cancer subtypes

	Cases		Controls		Model 1*		Cases		Controls		Model 2^	
	*N*	%	*N*	%	OR	95% CI	*N*	%	N	%	OR	95% CI
Alcohol consumption	All Invasive Breast Cancer											
< 7 drinks/week	**565**	**79.0**	**1876**	**86.3**			505	78.8	1690	86.3		
≥ 7 drinks/week	**150**	**21.0**	**298**	**13.7**	**1.27**	**1.01–1.61**	136	21.2	269	13.7	1.30	1.00–1.68
*ER*+												
< 7 drinks/week	313	79.6	1876	86.3			289	79.0	1690	86.3		
≥ 7 drinks/week	80	20.4	298	13.7	1.25	0.93–1.67	77	21.0	269	13.7	1.33	0.98–1.82
*PR*+												
< 7 drinks/week	256	79.8	1876	86.3			235	79.4	1690	86.3		
≥ 7 drinks/week	65	20.2	298	13.7	1.23	0.90–1.68	61	20.6	269	13.7	1.25	0.89–1.76
*HER2*+												
< 7 drinks/week	89	78.1	1876	86.3			79	78.2	1690	86.3		
≥ 7 drinks/week	25	21.9	298	13.7	1.48	0.92–2.38	22	21.8	269	13.7	1.57	0.92–2.68
*Non triple negative*												
< 7 drinks/week	366	78.7	1876	86.3			336	78.7	1690	86.3		
≥ 7 drinks/week	99	21.3	298	13.7	1.31	1.00–1.72	91	21.3	269	13.7	1.34	1.00–1.80
*ER*−												
< 7 drinks/week	**211**	**76.7**	**1876**	**86.3**			**183**	**77.5**	**1690**	**86.3**		
≥ 7 drinks/week	**64**	**23.3**	**298**	**13.7**	**1.57**	**1.13–2.19**	**53**	**22.5**	**269**	**13.7**	**1.5**	**1.01–2.14**
*PR*−												
< 7 drinks/week	**268**	**77.7**	**1876**	**86.3**			**237**	**78.0**	**1690**	**86.3**		
≥ 7 drinks/week	**77**	**22.3**	**298**	**13.7**	**1.5**	**1.10–2.01**	**67**	**22.0**	**269**	**13.7**	**1.50**	**1.07–2.10**
*HER2*−												
< 7 drinks/week	363	79.3	1876	86.3			327	79.0	1690	86.3		
≥ 7 drinks/week	95	20.7	298	13.7	1.26	0.95–1.66	87	21.0	269	13.7	1.32	0.97–1.79
*Triple negative*												
< 7 drinks/week	128	78.5	1876	86.3			108	77.7	1690	86.3		
≥ 7 drinks/week	35	21.5	298	13.7	1.5	0.96–2.22	31	22.3	269	13.7	1.5	0.93–2.40

*Model 1 adjusted for age (10 year categories) and study

^Model 2 adjusted for Model 1 covariates + education, age at menarche, BMI, parity, smoking status, menopause, and duration of oral contraceptive use

In the genetic main effects meta-analysis, we identified multiple testing-corrected statistically significant per-allele associations (see Additional file 1: Table S3, for locus-specific significance thresholds) between rs79865122-C in the *CYP2E1* locus on chromosome 10 and odds of ER- (OR (95% CI) = 0.21 (0.12, 0.40), *p*_*SNP*_ = 9.91 × 10^–7^), PR- (OR (95% CI) = 0.27 (0.15, 0.50), *p*_*SNP*_ = 1.87 × 10^–5^), and triple negative breast cancer (OR (95% CI) = 0.23 (0.11, 0.48), *p*_*SNP*_ = 1.15 × 10^–4^). -. This variant was also significantly associated with odds of ER- and PR- breast cancer using the joint test [ER- OR_≥7dpw_ (95% CI) = 3.92 (0.28, 55.41), OR_<7dpw_ = 0.24 (0.13, 0.44), *p*_*joint*_ = 3.74 × 10^−6^; PR- OR_≥7dpw_ (95% CI) = 1.77 (0.29, 10.87), OR_<7dpw_ (95% CI) = 0.31 (0.17, 0.55), *p*_*joint*_ = 9.52 × 10^–5^] (Additional file 1, Table 2). We also found a statistically significant SNP*alcohol consumption interaction between triple negative breast cancer and rs3858704-A in the *ALDH2* locus on chromosome 12 [OR_int_ (95% CI) = 6.28 (2.50,15.73), *p*_*int*_ = 8.97 × 10^–5^]. Stratified analysis of the *ALDH2* interaction revealed an increased odds of triple negative breast cancer in women who consumed ≥ 7 drinks per week (OR (95% CI) = 4.41 (1.79, 10.86), *p* = 1.26 × 10^–3^), and decreased odds in women who consumed < 7 drinks per week (OR (95% CI) = 0.57 (0.36, 0.89), *p* = 0.013). These associations were driven by Phase 1, as rs79865122 was filtered out of the Phase 2 results due to EffN_cases_ < 2, and rs3858704 was not present in Phase 2. Using LD Link, we identified rs78085062 as in LD with rs3858704 in 1000 Genomes AFR + EUR populations (r2 = 0.986). This variant was also not present in our Phase 2 results, nor were 6 other variants with r^2^ > 0.7. Significant results for each phase are presented in Additional file 1: Table S2.

**Table 2:** Summary of association results for loci with locus-wide significance for interaction

Hormone receptor status	CHR	POS	rsid	Nearest Gene	Alleles		EAF	≥ 7 drinks/week				< 7 drinks/week				Main and interaction effects										
					Effect	Other		OR snp	95%CI		Psnp	OR snp	95%CI		Psnp	Total *N*	EffN cases	OR snp	95% CI		Psnp	OR int	95% CI		Pint	Pjoint
ER-	10	135355763	rs79865122	*CYP2E1*	C	G	0.969	3.92	0.28	54.51	0.309	**0.24**	**0.13**	**0.44**	**3.22E-06**	1499	18.25	**0.21**	**0.12**	**0.40**	**9.91E-07**	15.98	1.38	185.25	0.027	**3.74E-06**
PR-							0.966	1.77	0.29	10.87	0.540	**0.31**	**0.17**	**0.55**	**7.24E-05**	1547	20.21	**0.27**	**0.15**	**0.50**	**1.87E-05**	5.30	0.89	31.62	0.067	**9.52E-05**
TripleNeg	12	111705893	rs3858704	*ALDH2*	A	G	0.271	4.41	1.79	10.86	1.26E-03	0.57	0.36	0.89	0.013	1392	34.88	0.58	0.37	0.91	0.017	**6.28**	**2.50**	**15.73**	**8.97E-05**	4.18E-04

We also examined known variants and proxies with r^2^ ≥ 0.8 in four alcohol metabolism gene regions (*ADH, CYP2E1*, *ALDH1,* and *ALDH2*) in our meta-analysis results for main genetic effects on invasive breast cancer. For each known variant extracted from the literature, we identified proxies in 1000 Genomes Phase 3 African and European populations using LD Link [52], and then extracted results for those known variants or their proxies from our meta-analysis of invasive cases. The variant or proxies with the lowest *p-*value are reported in Additional file 1, Table 3. For the *ADH* region on chromosome 4, we identified 14 known variants. Of those 14 known variants or proxies, rs2075633 was nominally significant in our meta-analysis results and directionally consistent in both phases [OR_SNP_ (95% CI) = 1.37 (1.06, 16.85), *p*_*SNP*_ = 0.016). Known variants rs1229984, rs2066702 and rs698 in the *ADH* region were not present in AMBER, but we were able to identify proxies with r^2^ ≥ 0.8 in 1000 Genomes Phase 3 African ancestry populations for rs2066702 and rs698 in our data, though the associations were not statistically significant. In the *ALDH1* region on chromosome 9, we identified 9 known variants previously examined for interaction effects of alcohol *mortality risk after breast cancer diagnosis [53]. None of these were statistically significant in our results. For the *CYP2E1* region on chromosome 10, we identified three previously reported variants, but none were significantly associated with odds of invasive breast cancer in our sample. For rs2031920, this is consistent with some previous studies [16, 54]. One known variant in the *ALDH2* region of chromosome 12, rs671, was not present in AMBER, and there were no proxies with r^2^ ≥ 0.08 in our data.

**Table 3:** Lookups of known alcohol risk variant (or proxies from 1000 Genomes Phase 3 AFR populations) associations with invasive breast cancer in the AMBER cohort

Gene region	Known variant	Proxy*	Chr	Pos (GRCh37)	*R*2	*D*'	Effect Allele	Other Allele	OR snp	95% CI		*P*-value	Direction	HetISq	HetPVal	Total *N*	EffN case	Reference PMID
ADH	rs1159918	–	4	100243009	1	1	A	C	1.170	0.971	10.886	0.099	++	0	0.770	2885	257.97	30052783
	rs1662033	–	4	100258381	1	1	T	G	0.876	0.689	6.292	0.279	-+	52.5	0.147	2885	150.97	30052783
	rs1693439	–	4	100245489	1	1	A	G	1.027	0.673	9.298	0.901	+?	0	1.000	1842	42.47	30052783
	*rs2075633*	–	*4*	*100238998*	*1*	*1*	*T*	*C*	*1.374*	*1.060*	*16.853*	*0.016*	*++*	*0*	*0.580*	*2885*	*132.07*	*30052783*
	rs283415	–	4	100270607	1	1	T	C	0.933	0.747	6.964	0.536	-?	0	1.000	1842	175.24	30052783
	rs3114046	–	4	100257432	1	1	T	C	1.022	0.669	9.202	0.920	+ ?	0	1.000	1842	42.39	30052783
	rs3811802	–	4	100244221	1	1	A	G	0.951	0.800	7.034	0.563	- +	67.4	0.080	2885	280.92	30052783
	rs4147536	–	4	100239112	1	1	A	C	1.118	0.940	9.763	0.207	++	0	0.580	2885	291.48	30052783
	rs4147542	–	4	100268553	1	1	T	C	0.891	0.747	6.279	0.202	−−	0	0.599	2885	287.70	30052783
	rs4699741	–	4	100278697	1	1	T	C	0.708	0.470	4.938	0.099	−−	63.4	0.098	2885	53.96	30052783
	rs9307239	–	4	100246937	1	1	T	C	1.015	0.848	8.020	0.869	++	0	0.924	2885	276.91	30052783
	rs2066702	–	4	100229017	1	1	G	A										26036284
	rs2066702	rs150627184	4	100163386	0.976	0.995	CT	C	1.494	0.867	24.674	0.148	?+	0	1.000	1043	31.16	30482948
	rs1229984	no proxies	4	100239319	1	1	G	A										12189594, 22331481
	rs698	–	4	100260789	1	1	C	T										30052783, 10530606
	rs698	rs13125415	4	100270452	0.834	1	A	G	1.438	0.880	21.537	0.148	?+	0	0.000	1043	38.29	22652248
ALDH1	rs168351		9	75517311	1	1	A	G	0.926	0.620	7.532	0.706	−?	0	1.000	1842	53.59	29190005
	rs1888202		9	75519251	1	1	C	G	1.133	0.905	10.336	0.277	+?	0	1.000	1842	162.23	29190005
	rs348463		9	75547612	1	1	T	C	0.971	0.622	8.425	0.898	?−	0	1.000	1043	45.23	29190005
	rs1424482		9	75563557	1	1	T	C	0.971	0.641	8.288	0.889	?−	0	1.000	1043	55.68	29190005
	rs722921		9	75544299	1	1	A	T	0.976	0.805	7.477	0.807	−+	0	0.565	2885	233.57	29190005
	rs63319		9	75524784	1	1	T	G	1.060	0.679	10.021	0.798	?+	0	1.000	1043	52.56	29190005
	rs7027604		9	75554952	1	1	A	C	1.155	0.718	12.270	0.553	?+	0	1.000	1043	43.75	29190005
	rs348481		9	75514436	1	1	C	T	0.981	0.635	8.542	0.932	?−	0	1.000	1043	47.98	29190005
	rs348461		9	75545070	1	1	A	T	0.941	0.793	6.911	0.492	−+	0	0.648	2885	290.75	29190005
CYP2E1	rs2031920	–	10	135339845	1	1	C	T	0.990	0.382	11.305	0.983	−?	0	1.000	1842	9.81	28074086, 12563175
	rs3813867	–	10	135339605	1	1	G	C	1.019	0.674	9.092	0.930	−+	22.4	0.256	2885	55.47	28074086
	rs6413432	–	10	135348544	1	1	T	A	1.233	0.850	13.563	0.269	++	0	0.752	2885	63.72	28074086
ALDH2	rs671	No proxies	12	112241766	1	1	G	A										32300124

*Proxy for known variant with the lowest p-value in our invasive cases meta-analysis genetic main effects results

## Discussion

Alcohol consumption has been associated with a moderately increased risk of breast cancer in women [2]. In addition, variants in genes involved in alcohol metabolism, such as *ADH* and *ALDH*, have been associated with an increased risk of cancer, including breast cancer, in some studies [2]. Previous investigations of alcohol gene and gene-exposure interaction have been primarily based on studies of individuals of European or Asian ancestry [9, 13, 23–32]. Our analysis of ethanol metabolism pathway genetic variants and SNP*alcohol consumption interactions and the odds of breast cancer was based on the African American Breast Cancer Epidemiology and Risk (AMBER) Consortium, a collaboration among four of the largest epidemiologic studies of breast cancer in African American women.

In the present analysis, we identified a positive association of consumption of ≥ 7 drinks per week with odds of all invasive breast cancer, as well as of ER and PR- breast cancer. The interaction of rs3858704-A in the *ALDH2* region of chromosome 12 with consumption of ≥ 7 drinks per week was significant in Phase 1, but neither that variant nor any proxies with r^2^ ≥ 0.7 were available in the Phase 2 data (Additional file 1: Table S2).We found a previously unreported association of rs79865122-C on chromosome 10 near *CYP2E1* with odds of ER- and PR- breast cancer, including a statistically significant joint main plus interaction effect. *CYP2E1* has been shown to have a lesser contribution to ethanol metabolism [55] than *ADH or ALDH*. At high ethanol concentrations, however, the ADH pathway becomes saturated, and activity of the microsomal ethanol oxidizing system (MEOS) pathway increases. As part of the MEOS, CYP2E1 metabolizes ethanol, and the process yields free radicals leading to oxidative stress [55]. In addition, elevated ethanol levels can interfere with the ability of *CYP2E1* to metabolize other substrates, such as medications, resulting in reduced clearance and elevated drug concentrations [55]. Multiple polymorphisms of *CYP2E1* have been identified, some of which are rare in populations of European ancestry, and some appear to have functional consequences on ethanol metabolism [11, 16]. One *CYP2E1* restriction fragment length polymorphism (RFLP) (CYP2E1*1D) has been shown to have a higher prevalence in women of African ancestry, and functional impact on in vivo metabolic activity in the presence of exposures known to increase expression of *CYP2E1* [obesity or recent (within 72 h) alcohol consumption] [56]. We were unable to locate any previous publications including a specific analysis of alcohol metabolism gene and gene-alcohol consumption effects on breast cancer risk among Black women [9, 10, 14, 15, 17].

Our analysis focused on the four major human alcohol metabolism genes or gene clusters, including *ADH, CYP2E1*, *ALDH1,* and *ALDH2.* Variants in these genes have been previously investigated for influencing ethanol metabolism or modifying the effect of alcohol on breast cancer risk, including ADH1B*2 (rs1229984), ADH1B*3 (rs2066702), ADH1C*1 (rs698), CYP2E1*5 (rs2031920-T, rs3813867-C, and rs6413432-A), CYP2E1*6 (rs6413432-A) and ALDH2*2 (rs671) [53, 57]. Some of the variants show population frequency differences, for example, East Asian populations have a higher frequency of ADH1B*2 (rs1229984-T) and ALDH2*2 (rs671-A) than other populations [58]. The ADH1B*2 (rs1229984) and ALDH2*2 (rs671) variants are fixed or very low frequency (minor allele frequency (MAF) ≤ 1%) in 1000 Genomes Phase 3 populations of African ancestry, while the ADH1B*3 (rs2066702) and ADH1C*1 (rs698) variants are common (MAF 6.94–28.24%) in these groups. In our lookups of known variants with odds of invasive breast cancer in our meta-analysis results, we replicated the association at rs2075633 in the *ADH* region on chromosome 4, which was nominally significant in our meta-analysis results and directionally consistent in both phases [OR_SNP_ (95% CI) = 1.37 (1.06, 16.85), *p*_*SNP*_ = 0.016).

This study had several strengths including a focus on breast cancer risk among African American women, breast cancer hormone receptor status information, the use of the MEGA array in Phase 2, comprehensive coverage of genomic regions containing ethanol metabolism genes and gene clusters, and examination of gene-alcohol interactions using both interaction and joint main effects + interaction models.

The analysis also had some limitations. Although the Phase 2 chip included variants designed to capture the genetic variation of global populations [59], compared to earlier genotyping chips that were designed to capture genetic variation in European populations, only 118 cases and 924 controls were genotyped on the Phase 2 chip, as opposed to 597 cases and 1250 controls on the Phase 1 chip. The Illumina Human Exome Beadchip v1.1 was used for Phase 1 genotyping, resulting in fewer SNPs included in those analyses compared to Phase 2 (18,792 variants in Phase 1 versus 46,519 variants in Phase 2). In this analysis, we also focused primarily on genetic factors and have not placed this work in the context of multilevel determinants of breast cancer risk, including social determinants and structural racism [60–63]. Exposure status and information on other covariates were self-reported, which would increase the possibility of recall bias in the case-controls studies, but not in the prospective BWHS. In addition, the number of women who reported consuming ≥ 7 drinks/week was limited (150 cases and 298 controls). This is consistent with reported exposure patterns, notably the lower frequency of heavy drinking in Black women compared to white women [64], which may have influenced our power in this study relative to similarly sized studies of white women (see Additional file 2). Finally, while we attempted to account for many other variables that may influence both breast cancer risk and alcohol consumption, including age at menarche, parity, menopausal status, BMI, smoking behavior, and use of oral contraceptives, others, such as diet and physical activity, were not included in our analysis.

## Conclusions

In the present study, we examined the relationship between genetic variation in key ethanol metabolism genes with odds of breast cancer. We also evaluated interactions between alcohol intake and genetic variation on odds of breast cancer. As has been reported in a previous AMBER analysis [3], we identified a significant association between consumption of ≥ 7 drinks per week and ER- and PR- breast cancer in our minimally adjusted models, and the associations remained significant in our fully adjusted models. We found a statistically significant joint association with rs79865122 in the *CYP2E1* region of chromosome 10 with ER- and PR- and breast cancer, potentially pointing to genomic regions influencing the associations identified in our exposure-outcome models examining the impact of consumption of ≥ 7 drinks per week on odds of breast cancer. Although we used the largest available genetics resource relevant to breast cancer in Black women, additional research to further validate the interaction of genetic variants in alcohol metabolism genes and alcohol consumption on odds of breast cancer is warranted.

## Supplementary Information

**Additional file 1**. **Table S1**: Characteristics of AMBER analytic sample participants by study and phase;** Table S2**. Significant main, interaction, and joint (2df) effects of SNP and alcohol intake on breast cancer hormone receptor subtypes in AMBER by phase;  **Table S3**: Statistical significance thresholds by phase in AMBER.

**Additional file 2**. Power plots. **Fig. S1**: Power to detect main genetic effects for minor allele frequencies= 0.01–0.25. OR = 1.5–4.5 in: **A** Phase 1 sample size 1847, **B** Phase 1 sample size 1392, and alpha = 6.79E-5; **C** Phase 2 sample size 1043, **D** Phase 2 sample size 950, and alpha = 1.32E-4; and **E** meta-analysis sample size 2889, **F** sample size 2494, and alpha = 1.50E-4. **Fig. S2**: Power to detect interaction effects in **A** Phase 1: N = 1800, case proportion = 32%, alpha = 6.79E-5; **B** Phase 2: N = 1000, case proportion = 12%, alpha=1.32E-4; **C** meta-analysis: N = 2900, case proportion = 25%, alpha=1.50E-4. OR_G = genetic main effect, OR_GE = G*E interaction effect.

## Data Availability

The data supporting the conclusions of this article are available in dbGaP (authorized access required, Study Accession: phs000669.v1.p1, https://www.ncbi.nlm.nih.gov/projects/gap/cgi-bin/study.cgi?study_id=phs000669.v1.p1).

## Abbreviations

ADH: Alcohol dehydrogenase
ALDH: Aldehyde dehydrogenase
AMBER: African American Breast Cancer Epidemiology and Risk Consortium
AOR: Adjusted odds ratio
BMI: Body mass index
BWHS: Black Women’s Health Study
CAT: Catalase
CBCS: Carolina Breast Cancer Study
CIDR: Center for Inherited Disease Research
CYP2E1: Cytochrome P450 2E1
DCIS: Ductal carcinoma in situ
ER: Estrogen receptor
FGFR2: Fibroblast growth factor receptor 2
GAC: Genetic Analysis Center
GEE: Generalized estimating equations
GWAS: Genome-wide association study
HER2: Human epidermal growth factor receptor 2
MAC: Minor allele count
MAF: Minor allele frequency
MEGA: Multi-Ethnic Global Array
MEOS: Microsomal ethanol oxidizing system
OR: Odds ratio
PLCO: Prostate, Lung, Colorectal and Ovarian Cancer Screening Trial
PR: Progesterone receptor
QC: Quality control
SNP: Single nucleotide polymorphism
WCHS: Women’s Circle of Health Study
