# Supplementary material for: Influence of alcohol consumption and alcohol metabolism variants on breast cancer risk among Black women: results from the AMBER consortium
Source: Breast Cancer Res. 2023 Jun 12;25:66. doi: 10.1186/s13058-023-01660-1 (PMC10259046; doi:10.1186/s13058-023-01660-1)
Supplement: Supplementary file 2 — Additional file 2. Power plots. Fig. S1: Power to detect main genetic effects for minor allele frequencies= 0.01–0.25. OR = 1.5–4.5 in: A Phase 1 sample size 1847, B Phase 1 sample size 1392, and alpha = 6.79E-5; C Phase 2 sample size 1043, D Phase 2 sample size 950, and alpha = 1.32E-4; and E meta-analysis sample size 2889, F sample size 2494, and alpha = 1.50E-4. Fig. S2: Power to detect interaction effects in A Phase 1: N = 1800, case proportion = 32%, alpha = 6.79E-5; B Phase 2: N = 1000, case proportion = 12%, alpha=1.32E-4; C meta-analysis: N = 2900, case proportion = 25%, alpha=1.50E-4. OR_G = genetic main effect, OR_GE = G*E interaction effect. [file 13058_2023_1660_MOESM2_ESM.pdf]

Power

We estimated power to detect the main effects of SNPs as well as power to detect gene-environment interaction effects in each phase and the combined meta-analysis assuming an additive genetic model for SNPs with MAF between 0.01-0.25, OR_{SNP} between 1.5-4.5, odds ratio for the exposure (OR_E) of 1.5, and OR_{INT} between 0.05-0.2, using the genpwr R package [1](Figures S1-S2, Additional file 2). In Phase 1, at our most conservative Phase 1 locus-specific significance threshold (0.05/number of independent variants per locus) of 6.79×10^{-5} for the *ADH* region on chromosome 4, given our maximum sample size of 597 cases and 1,250 controls, we have 80% power to detect main genetic effects with an $OR_{SNP} \geq 1.5$ at MAF = 0.22. For $OR_{SNP} \geq 2.5$, we have 93% power to detect main genetic effects at MAF = 0.04. In Phase 2, given our maximum sample size of 118 cases and 924 controls, using our most conservative Phase 2 locus-specific significance threshold of 1.32×10^{-4} for the *ADH* region on chromosome 4, we have 86% power to detect main genetic effects with $OR_{SNP} \geq 2.5$ at MAF = 0.12. For interaction effects, with a proportion of the population who have the environmental exposure of 20% [2], given our maximum sample size of 715 cases and 2,174 controls, alpha = 0.00015, $OR_{alcohol} = 1.5$, and $OR_{SNP} \geq 2.5$, we have 80% power to detect $OR_{INT} = 0.2$ at MAF = 0.05, and 82% power to detect $OR_{INT} = 0.1$ at MAF = 0.03. For $OR_{SNP} = 1.5$, holding the other parameters constant, our power to detect $OR_{INT} = 0.2$ at MAF = 0.05 drops to 64%, and our power to detect to $OR_{INT} = 0.1$ at MAF = 0.03 drops to 62%.

Supplemental References

1. Moore CM, Jacobson SA, Fingerlin TE. Power and Sample Size Calculations for Genetic Association Studies in the Presence of Genetic Model Misspecification. Hum Hered. 2020;84:256–71.

2. Solomon CA, Laditka SB, Forthofer M, Racine EF. Black-white disparities in alcohol consumption trends among women in the United States, 1990–2015. J Ethn Subst Abuse. 2019;20:1–22.

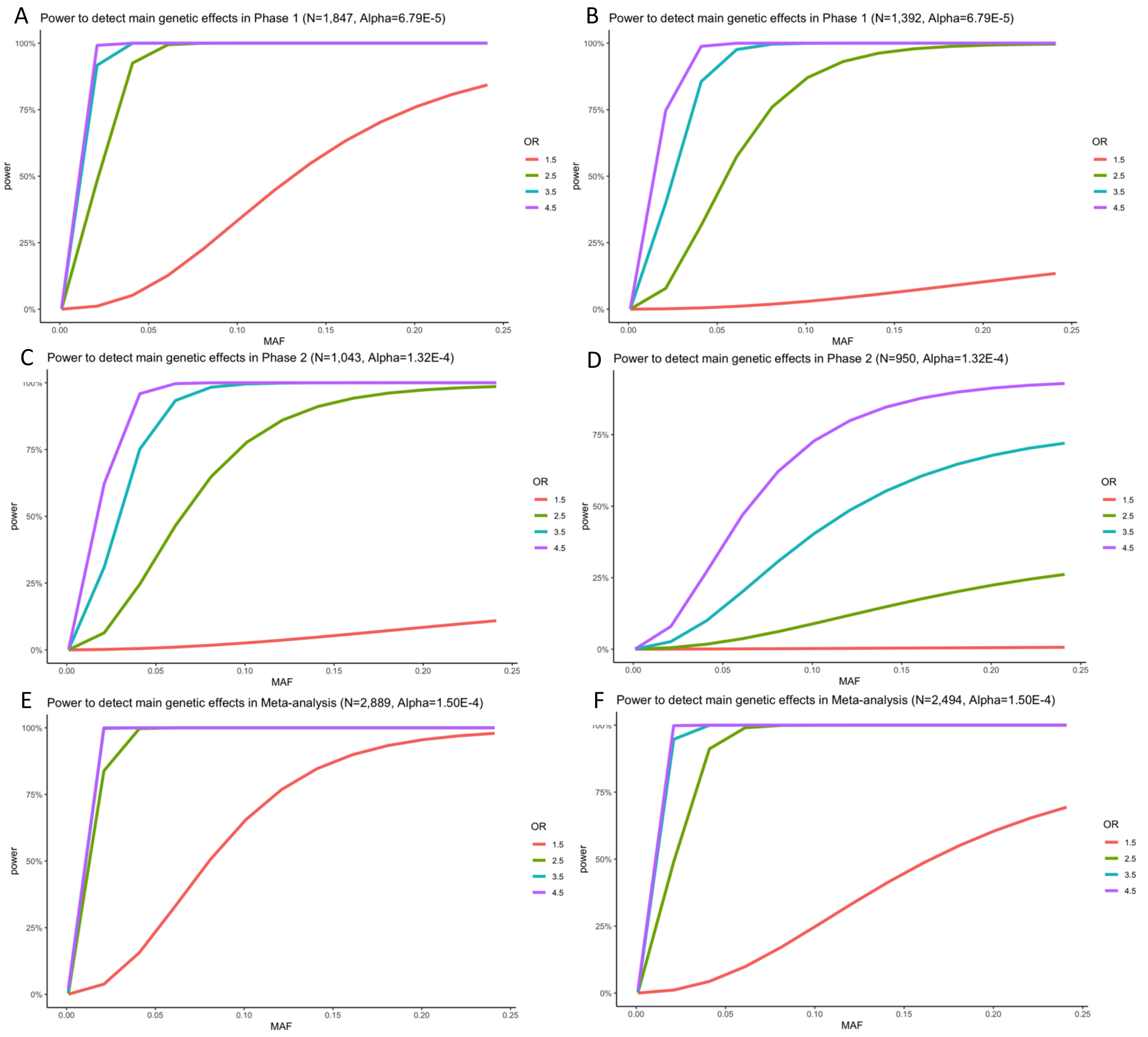

Figure S1. Power to detect main genetic effects (ORs =1.5-4.5) in Phase 1 for samples sizes N=1,847 (Panel A) and N=1,392 (Panel B) at alpha = 6.79 x 10^{-5}. Power to detect main genetic effects (ORs =1.5-4.5) in Phase 2 for samples sizes N=1,043 (Panel C) and N= 950 (Panel D) at alpha = 1.32 x 10^{-4}. Power to detect main genetic effects (ORs =1.5-4.5) in meta-analyses for samples sizes N=2,889 (Panel E) and N=2,494 (Panel F) at alpha = 1.50 x 10^{-4}.

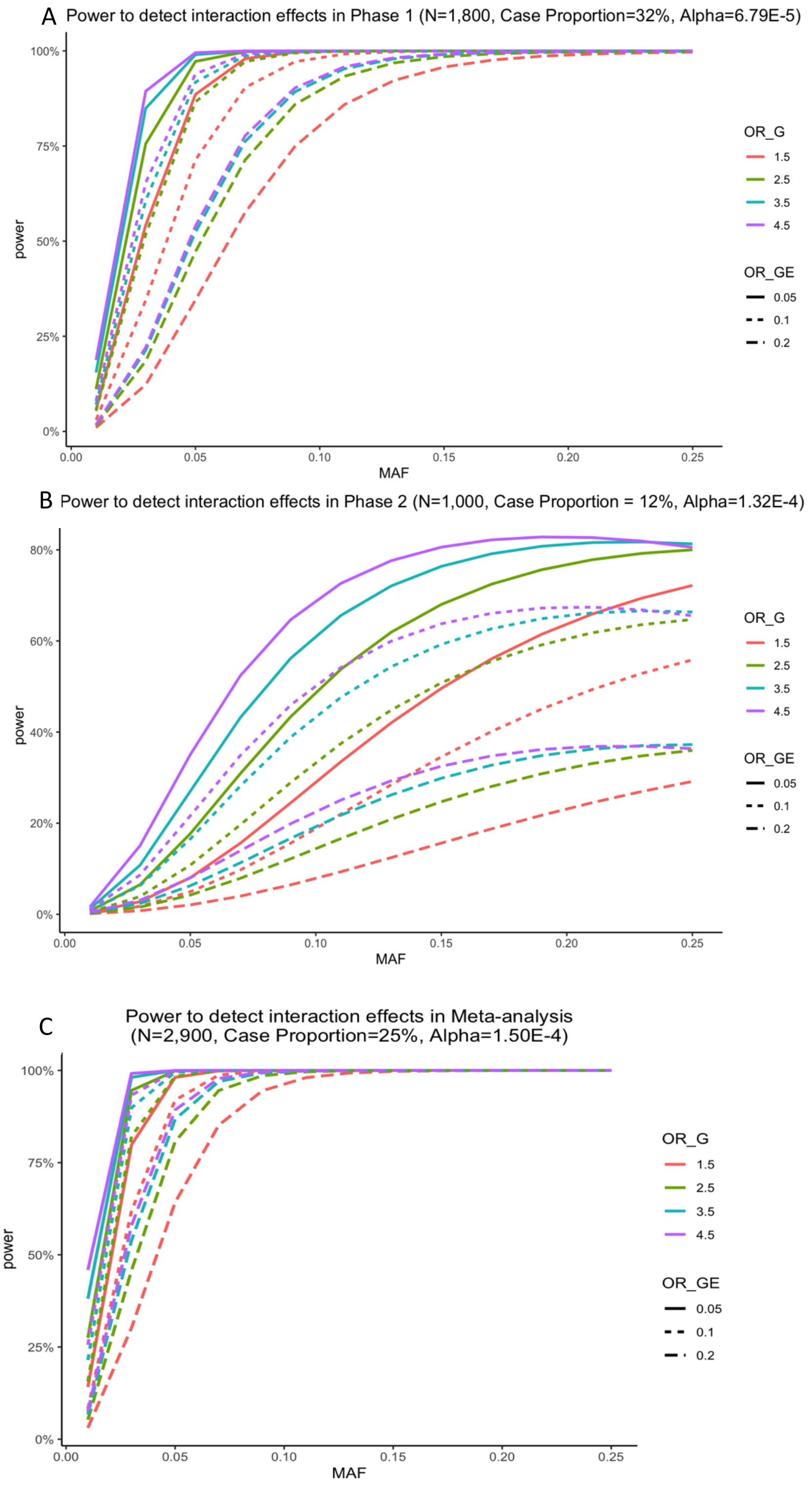

Figure S2. Power to detect interaction effects given main effects ORs =1.5-4.5 and interaction effects=0.05-0.2 in Phase 1 (Panel A: N=1,800, case proportion=32%, at alpha = 6.79 x 10^{-5}), Phase 2 (Panel B: N=1,000, case proportion = 12%, at alpha = 1.32 x 10^{-4}), and meta-analysis (Panel C: N=2,900, case proportion = 25%, at alpha = 1.50 x 10^{-4}).
